# The mesenchymal stem cells in multiple sclerosis (MSCIMS) trial protocol and baseline cohort characteristics: an open-label pre-test: post-test study with blinded outcome assessments

**DOI:** 10.1186/1745-6215-12-62

**Published:** 2011-03-02

**Authors:** Peter Connick, Madhan Kolappan, Rickie Patani, Michael A Scott, Charles Crawley, Xiao-Ling He, Karen Richardson, Kelly Barber, Daniel J Webber, Claudia AM Wheeler-Kingshott, Daniel J Tozer, Rebecca S Samson, David L Thomas, Ming-Qing Du, Shi L Luan, Andrew W Michell, Daniel R Altmann, Alan J Thompson, David H Miller, Alastair Compston, Siddharthan Chandran

**Affiliations:** 1 Dept. of Clinical Neurosciences, University of Cambridge, UK; 2 Anne MacLaren Laboratory for Regenerative Medicine, University of Cambridge, UK; 3 NMR Research Unit, Dept. of Neuroinflammation, UCL Institute of Neurology, London, UK; 4 Blood & Marrow Transplant Unit, Addenbrooke's Hospital, Cambridge, UK; 5 The Advanced Magnetic Resonance Imaging Group, University College London, London, UK; 6 Dept. of Pathology, University of Cambridge, UK; 7 Medical Statistics Unit, London School of Hygiene and Tropical Medicine, UK; 8 Dept of Brain Repair & Rehabilitation, University College London, UK; 9 Euan MacDonald Centre, University of Edinburgh, UK

## Abstract

**Background:**

No treatments are currently available that slow, stop, or reverse disease progression in established multiple sclerosis (MS). The Mesenchymal Stem Cells in Multiple Sclerosis (MSCIMS) trial tests the safety and feasibility of treatment with a candidate cell-based therapy, and will inform the wider challenge of designing early phase clinical trials to evaluate putative neuroprotective therapies in progressive MS. Illustrated by the MSCIMS trial protocol, we describe a novel methodology based on detailed assessment of the anterior visual pathway as a model of wider disease processes - the "sentinel lesion approach".

**Methods/design:**

MSCIMS is a phase IIA study of autologous mesenchymal stem cells (MSCs) in secondary progressive MS. A pre-test : post-test design is used with healthy controls providing normative data for inter-session variability. Complementary eligibility criteria and outcomes are used to select participants with disease affecting the anterior visual pathway.

**Results:**

Ten participants with MS and eight healthy controls were recruited between October 2008 and March 2009. Mesenchymal stem cells were successfully isolated, expanded and characterised *in vitro *for all participants in the treatment arm.

**Conclusions:**

In addition to determining the safety and feasibility of the intervention and informing design of future studies to address efficacy, MSCIMS adopts a novel strategy for testing neuroprotective agents in MS - the *sentinel lesion approach *- serving as proof of principle for its future wider applicability.

**Trial registration:**

ClinicalTrials.gov (NCT00395200).

## Background

Multiple sclerosis (MS) is the commonest neurological cause of disability in young adults, affecting over 1.3 million people worldwide. It is a chronic multifocal and multiphasic immune mediated disorder characterised pathologically by inflammatory demyelination, axonal injury and partial remyelination [1]. Although recent evidence suggests that conventional disease modifying approaches can mitigate demyelination and secondary axonal loss resulting from focal inflammation if given during a narrow therapeutic-window in nascent RR-MS,[2,3] there are currently no therapies that slow, stop, or reverse progressive axonal loss in established disease. Mesenchymal stem cells (MSCs) are recognised as a candidate in this respect due to evidence that they promote oligodendrogenesis both *in vitro *and *in vivo*,[4,5] result in functional improvement in animal models of MS,[6,7] and confer benefit in non-neurological T-cell driven autoimmune human disease [8].

Trial design for the assessment of putative neuroprotective agents in MS presents a range of challenges including the need to identify patients who will benefit from treatment as well as to provide information on the mode of action of any intervention [9]. This requires clinical trial protocols that differ from those used in the evaluation of disease-modifying therapies in terms of both participant selection and measurement(s) of efficacy. Current methods for detecting neuroprotection are limited to comparatively insensitive assessments of overall response. These composite measures risk failure to detect subtle but nonetheless meaningful effects of intervention on components of the disease process that drive cumulative disability. Therefore, neuroprotective trial design must aim to select outcomes that are informative with respect to stages of the disease other than the relapsing-remitting phase, and which demonstrate the effects of targeting both immunological and neurobiological components of the complex pathogenesis. This requires a combination of clinical outcome measures to register improved function in addition to the use of paraclinical observations and novel biomarkers that can inform on the mechanisms of therapeutic effect and detect benefits that lie below clinical thresholds. Against this background, we report the design and baseline cohort characteristics of a phase IIA trial of autologous MSC therapy as a putative neuroprotective therapy for secondary progressive MS that uses novel approaches to address these challenges. By providing a detailed methodological description, we aim to inform the development of trial design in the wider setting of neuroprotective therapies for progressive MS.

## Methods

### Trial design

The MSCIMS Trial uses an 18-month pre-test : post-test design with a single treatment of autologous mesenchymal stem cells at 12 months. A parallel cohort of normative controls was also recruited to determine inter-session variability of assessment methods.

### Trial Centres

The trial was conducted over two sites. Clinical assessments were based at the Wellcome Trust Clinical Research Facility, Cambridge, UK; imaging assessments were carried out at the UCL Institute of Neurology, London, UK.

### Trial objectives

MSCIMS aims to establish the safety and feasibility of autologous intravenous mesenchymal stem cell therapy in multiple sclerosis (phase IIA). The primary objective is to describe the safety profile over six months of intravenously administered autologous MSCs at a dose of 1 - 2 × 10^6 ^cells / kg in patients with multiple sclerosis. The secondary objective is to explore the potential efficacy over six months of intravenously administered autologous MSCs at a dose of 1 - 2 × 10^6 ^cells / kg by clinical, neurophysiological and imaging assessments.

### Participant Selection

Patients were selected on the basis of having secondary progressive MS and prior involvement of the afferent visual pathway (table 1). Given its use as an outcome measure, a lower limit for retinal nerve fibre layer (RNFL) thickness was imposed to avoid a floor effect.

**Table 1:** Eligibility criteria

Clinically definite multiple sclerosis
Expanded Kurtzke Disability Status Score (EDSS) 2.0 - 6.5 inclusive
Clinical evidence of optic nerve involvement on history or examination*
Abnormal visual evoked potential from one or both eyes suggestive of demyelination
Retinal nerve fibre layer (RNFL) thickness of at least 45 microns in one eye
T_2 _lesion on MRI of optic nerve
Age 18 - 65 inclusive
Capacity to give consent
No serious underlying bleeding disorder
Not on Beta-interferon or Glatiramer acetate within six months of trial entry and not previously on other disease modifying therapies at any point

* Defined as history of optic neuritis, Unthoff's phenomenon, or optic atrophy on examination

### Recruitment

Participants were recruited from secondary care referrals in the East Anglia and North-London regions of the UK. Referral criteria were: clinically definite multiple sclerosis; Expanded Kurtzke Disability Status Score (EDSS) 2.0 - 6.5 inclusive; clinical evidence of optic nerve involvement; not on Beta interferon or Glatiramer acetate within 6 months of referral, and not previously on other disease modifying therapies at any point.

### MSC isolation, expansion, characterisation & pre-administration safety checks

In compliance with JACIE (Joint Accreditation Committee ISCT [International Society for Cellular Therapy] - EBMT [European Group for Blood and Marrow Transplantation]) requirements, normal serology was confirmed for HIV 1 & 2, Hepatitis B, Hepatitis C, HTLV 1 & 2, Syphilis, and CMV less than thirty days before bone marrow aspiration. Following demonstration of normal platelet count, PT and APTT, bone marrow aspiration was performed under local anaesthesia and sedation (with anaesthetic supervision) in an operating theatre using standard techniques.

Clinical-grade mesenchymal stem cells were generated under good manufacturing practice conditions using standard operating procedures based on the European Group for Blood and Bone Marrow Transplantation developmental committee [8]. Briefly, bone-marrow mononuclear cells were separated by density gradient centrifugation in Ficoll-Paque™ PREMIUM (GE Healthcare UK Ltd, UK) as previously described [10]. Washed cells were re-suspended in PBS/EDTA (Miltenyi Biotec Ltd, UK) and cultured in Dulbecco's modified Eagle's medium-low glucose (Invitrogen, UK) supplemented with 10% foetal bovine serum (Hyclone, Perbio Science, UK) and plated at a density of 1 × 10^8 ^cells per cell-factory (Nunc, Thermo Scientific, UK). Cultures were maintained at 37°C in a humidified atmosphere containing 5% CO_2 _in cell factories. When the cultures were near confluence (>80%), cells were detached by treatment with 0.25% trypsin-EDTA (Invitrogen, UK) and re-plated at 3.5 × 10^6 ^cells per cell factory. When 2 × 10^6 ^cells /kg (of participant) or more were obtained, MSCs were harvested and cryopreserved in 4.5% human albumin solution (BPL, UK) with Dimethyl Sulphoxide (Origen Biomedical Inc.) at a final concentration of 10%.

MSCs were characterised in accordance with International Society of Cellular Therapy (ISCT) recommendations [11]. Briefly, this included evidence of tri-lineage differentiation potential (adipocyte, chondrocyte, osteocyte) and flow cytometry assessment confirming expression of CD73, CD90, and CD105 surface molecules (>95%) and absence of CD34, CD45, CD14, and CD3 (< 2%). Release criteria of mesenchymal stem cells for clinical use included absence of contamination by pathogens (as documented by aerobic and anaerobic cultures and mycoplasma testing before release), and lack of any genomic copy number changes by 1Mb resolution BAC array comparative genomic hybridization (aCGH) performed in the Dept. of Pathology, University of Cambridge [12].

### Intervention dose, route, and administration procedures

Participants received one treatment with autologous MSCs administered intravenously at a dose of 1 - 2 × 10^6 ^cells/kg. Administration was performed as a day-case procedure following pre-medication with chlorpheniramine 10 mg, hydrocortisone 100 mg, and metoclopramide 10 mg. Cryopreserved MSCs were thawed (≤ 4 minutes) and immediately infused over 15 minutes. Administration (mean 167.2 ml, range: 89 to 246 ml) was followed by infusion of normal saline (500 ml) over 4 hours. Participants were monitored clinically for evidence of adverse reactions over a minimum of 4 hours.

### Outcome selection & assessment schedule

In order to increase power to detect efficacy through use of tailored outcomes, complementary eligibility criteria and outcome measures were selected allowing detailed assessment of participants with disease affecting the afferent visual pathway. Study of the entire visual pathway as a model of wider disease processes allows the use of clinical and paraclinical outcomes that can inform on aspects of both structure and function [13]. Participants were assessed at 12 (M-12) and 6 (M-6) months before treatment, immediately prior to treatment (M0), and at 3 (M3) and 6 (M6) months after treatment. Assessment at each time point was split into two visits with an interval of less than two weeks: clinical assessment and visual evoked potentials were performed in Cambridge, UK; MRI, optical coherence tomography (OCT), and neuro-ophthalmological assessments were performed in London, UK. In addition, patients were reviewed weekly on four occasions following treatment to assess immediate clinical response and monitor blood parameters including: full blood count, prothrombin time, accelerated partial thromboplastin time, erythrocyte sedimentation rate, serum urea & electrolytes, serum calcium and phosphate, serum albumin, bilirubin, alanine transaminase, aspartine transaminase. C-reactive protein, thyroid-stimulating hormone, complement C3 & C4, lymphocyte subsets, and serum IgG, IgA & IgM.

### Clinical outcome measures

Detailed neurological and medical history was obtained at screening and updated at each visit. Specific enquiry for adverse events was performed and participants encouraged to keep written records of interim events. Clinical outcomes assessed at each visit were: Multiple Sclerosis Impact Scale -- 29 (MSIS-29), Beck's Depression Inventory II (BDI-II), Addenbrooke's Cognitive Examination - Revised (ACE-R), Multiple Sclerosis Functional Composite (MSFC), and EDSS. Neuro-opthalmological outcomes assessed at each visit were: Visual acuity using a retro-illuminated EDTRS chart, Contrast Acuity using retro-illuminated Sloan charts, Colour vision using the Farnsworth-Munsell 100-Hue test, and Visual Fields by automated static perimetry (Humphrey field analyser, 30-2 protocol).

### Paraclinical outcome measures

Paraclinical outcome measures at each visit were: visual evoked responses (VER), measures of RNFL thickness and Macular Volume (MV) using optical coherence tomography (OCT), and MRI measures of brain and optic nerve. Whole and central field checkerboard pattern reversal VERs were recorded using reversal achromatic checks subtending 60' at the eye. OCT images were acquired with a time domain OCT (Stratus OCT Model 3000; Carl Zeiss Meditec, Dublin, CA, USA). All OCT imaging was performed by a single observer (MK). RNFL images were acquired by taking three circular 3.4-mm scans, centred on the optic disc, the mean of which was used to express RNFL thickness (Fast RNFL thickness protocol). The thicknesses of the quadrants of the RNFL were automatically calculated by the OCT device software. Macular thickness maps were acquired by making six radial scans centred on the fovea, and by construction of a map from these scans (Fast macular thickness map scanning protocol). OCT images are given a signal strength by the Stratus OCT device, with a maximum of 10. OCT images were rejected if an individual eye was < 7, the inter-eye signal strength difference was >2, or if the difference in signal strength between baseline and follow-up scans was >2.

### MR imaging methodology

MR images were acquired using a Siemens MAGNETOM 3.0T Tim Trio scanner (Siemens, Erlangen, Germany) with a twelve-element receiver head coil. Total acquisition time was approximately 75 minutes, extended to 130 minutes for the two visits involving optic nerve diffusion tensor imaging (DTI) and functional MRI (fMRI).

#### (i) Optic nerve imaging

Optic nerve lesions were identified using a fat saturated turbo spin echo sequences acquired at two different echo times: coronal oblique, TR = 2960 ms, TE1 = 12 ms, TE2 = 71 ms, number of averages = 4, matrix size = 512 × 384, field of view (FOV) = 24 × 18 cm^2^, in-plane resolution = 0.5 × 0.5 mm^2^, 16 × 3.0 mm slices for each, acquisition time = 4 minutes per sequence.

Optic nerve area was assessed using a fat saturated short echo fast fluid attenuated inversion recovery (sTE fFLAIR) sequence with the following parameters: TR = 1830 ms, TE = 13 ms, TI = 800 ms, matrix size = 384 × 306, 22 × 18 cm^2 ^FOV, in-plane resolution = 0.60 × 0.60 mm^2^, 16 × 3 mm contiguous coronal slices, acquisition time = 13 minutes. The mean cross sectional intra-orbital optic nerve area was calculated by averaging at least four slices of the intra-orbital segment, using semi-automatic contouring with manual correction as previously described [14].

Optic nerve magnetisation transfer ratio (MTR) was assessed by acquiring 3D gradient echo sequences with and without MT pre-pulse: TR = 36 ms, TE = 3.0 ms, number of averages = 2, flip angle = 12°, matrix size = 256 × 192, FOV = 19 × 14.25 cm^2^, in-plane resolution = 0.70 × 0.70 mm^2^, 60 × 1.5 mm contiguous coronal slices, acquisition time = 16 minutes. Optic nerves were contoured from chiasm to globe on the image acquired without the MT pre-pulse using a semi-automatic threshold based method with manual correction if required. MTR maps were calculated on a voxel by voxel basis and the contours transferred to the MTR maps, allowing calculation of MTR after manual correction for mis-registration due to movement between sequences with and without the MT pre-pulse.

Optic nerve diffusion tensor imaging (DTI) was performed by acquiring 3D fat and fluid attenuated spin echo single shot echo planar sequences: TR = 6 s, TE = 84 ms, TI = 1.2 s, matrix size = 128 × 64, FOV = 15 × 7.5 cm^2^, in-plane resolution = 1.17 × 1.17 mm^2^, 16 × 4 mm contiguous coronal oblique slices, diffusion gradients were applied in six directions with b = 600 s/mm^2 ^and one b = 0 image was also acquired, number of averages = 40, acquisition time = approximately 28 minutes [15]. The seven diffusion-weighted volumes (one b_0 _plus six b = 600 s/mm^2^), were then eddy-current corrected using the FSL software library http://www.fmrib.ox.ac.uk/fsl and diffusion metrics calculated using the Camino software package http://www.camino.org.uk[16]. The DT was calculated on a voxel by voxel basis. Square regions of interest (ROI) of fixed size (2 × 2 voxels [5.5 mm^2^]) were placed on the b_0 _image, guided by maximum signal intensity and minimum standard deviation. ROIs were then applied to the calculated parameter maps in order to determine quantitative diffusion indices. Mean diffusivity (MD), axial diffusivity (AD), radial diffusivity (RD), and fractional anisotropy (FA) were calculated by averaging parameters across at least three slices per optic nerve.

#### (ii) Brain

Axial T2-Proton density (PD) weighted dual echo turbo spin echo sequences were acquired: axial, TR = 3 seconds, TE1 = 11 ms, TE2 = 101 ms, matrix size = 192 × 256, FOV = 24 × 18 cm^2^, in-plane resolution = 0.9 × 0.9 mm^2^, 48 × 3 mm contiguous slices per echo (total 96 slices), acquisition time = 4 minutes. Hyperintense lesions were contoured from the PD image using a semi-automated threshold-based method, and cross-checked with contouring of T2 weighted images.

Axial T1 weighted spin echo sequences were also acquired: TR = 710 ms, TE = 8.5 ms, matrix size = 233 × 256, FOV = 22 × 22 cm^2^, in-plane resolution = 0.9 × 0.9 mm^2^, 48 × 3 mm contiguous slices, number of averages = 2, acquisition time = 5 minutes. T1 hypointense lesions were contoured using a semi-automated threshold based method.

Brain atrophy imaging was performed by 3D T1 weighted Modified Driven Equilibrium Fourier Transform (MDEFT) gradient echo sequences:[17-19] sagittal, TR = 7.13 ms, TE = 2.33 ms, matrix size = 224 × 256, FOV = 256 × 244 mm^2^, in-plane resolution = 1.0 × 1.0 mm^2^, 176 × 1 mm contiguous slices acquired in 12 minutes. Fully automated segmentation was performed for longitudinal assessment of atrophy using Structural Image Evaluation using Normalisation of Atrophy (SIENA); single time-point brain volumes were attained using SIENAX [20].

Brain MTR measures were obtained using coronal 3D gradient echo sequences with and without MT pre-pulse: TR = 26 ms, TE = 3.0 ms, flip angle = 10°, matrix size = 256 × 160, FOV = 25 × 16 cm^2^, in-plane resolution 1.0 × 1.0 mm^2^, 208 × 1.0 mm contiguous slices, acquisition time = 20 minutes in total. The MT images with and without pre-pulse, and the MDEFT T1 images were orientated to the axial plane, re-sliced, and registered to the PD-T2 image set. MTR maps were generated from the registered data and the registered 3D T1 volumetric image segmentation performed using SPM (Version 8)[21] with and without the lesions masked using the lesion contoured PD image. These extracted brain segments were applied to mask the MTR map into grey and white matter segments. These tissue MTR maps were then refined by applying a lower threshold of 10 pu, and erosions for whole brain & grey matter (1 voxel) and white matter (2 voxels), to remove partial volume voxels. T1 lesion masks were also applied to allow generation of MTR histograms for: whole brain (WB), grey matter (GM), white matter (WM), normal-appearing grey matter (NAGM) (GM with the lesions removed), normal appearing white matter (NAWM), T2/PD-lesions, and T1 hypointense lesions. MTR histograms in controls were obtained for: WB, GM, and WM.

Functional MR imaging was performed by acquiring T2* -weighted images depicting blood oxygen level dependent (BOLD) contrast: near axial (whole brain), TR = 3940 ms, TE = 30 ms, matrix size = 64 × 64, FOV = 192 mm, slice thickness = 3 mm. Four experiments of 5 minutes duration were performed using differing visual stimulation patterns as previously described [22]. Briefly, in a five-minute experiment, the subject uses binocular vision through red-green filter goggles to view a projected monitor while lying in the scanner. A reversing checkerboard pattern was shown for sixteen-second epochs of red or green stimuli, alternating with sixteen-second epochs of no stimulation. Red and green colour patterns were presented randomly in order to avoid predictability. The red-green filters were reversed by swapping the goggles between each five-minute session (*ie*. if the first run had red filter for the right eye, this was changed to green for the second run). Monocular stimulation was achieved for each epoch by matching the colour of stimulus and filter so that the eye looking through the red filter could only see the red checkerboard pattern while the other eye was unstimulated by red (and vice versa for green). Each five-minute experiment consisted of four epochs of green and four of red, thus stimulating both eyes four times each. Attention was monitored by a manual response task in the non-stimulation epochs. Analysis was performed using Statistical Parametric Mapping software (SPM 8.0, Wellcome Trust Centre for Neuroimaging, UCL, London, UK). Images were realigned, co-registered, and normalised to the T1 volumetric image obtained at that session. Images were then smoothed, and the experimental model was specified. Effects were analysed for each eye independently.

### Data & Safety Monitoring

An independent data monitoring committee was appointed following recruitment of the first participant and instructed to review progress 4 weeks after treatment of the first three participants and at six-month intervals thereafter.

### Blinding and data analysis

Optic nerve area, optic nerve MTR, and optic nerve DTI based outcomes were assessed by observers blind to participant status (pre/post-treatment). Lesional analysis was performed shortly after image acquisition at each visit. Brain volume, brain MTR, and fMRI analyses were performed using automated methods with minimal manual corrections; blinding was not therefore performed. Group comparisons for baseline outcomes were performed by Student's t-test. Following trial completion, safety data will be analysed to assess whether the post-intervention period is associated with an increase in adverse events; and efficacy data will be analysed to assess whether the post-intervention period is associated with a difference in pre-defined outcomes. No interim analyses are planned.

### Ethical approval and trial-registration

The MSCIMS trial was granted ethical approval following Research Ethics Committee review (07/Q0108/104) and is registered with the NIH clinical trials database (NCT00395200).

## Results

### Recruitment & retention of participants

**Figure 1:**
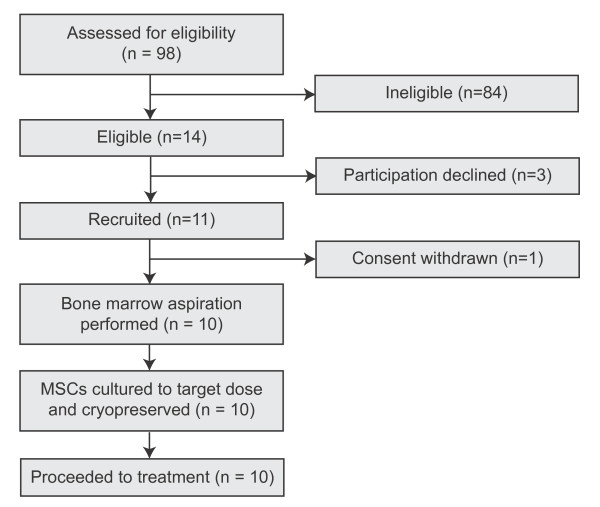
**Recruitment and retention to treatment cohort**.

Ninety-eight subjects were screened between November 2007 and June 2009, with fourteen (14.3%) meeting all eligibility criteria (Figure 1). Three subjects declined participation following detailed discussions and one participant withdrew consent shortly after recruitment for personal reasons.

### Participant characteristics

All participants in the treatment arm had secondary progressive MS with mean disease duration of 14.4 years (SD 7.9). Mean age was 48.8 years (SD 4.1), sex ratio was 3:7 (F:M) (table 2). Nine participants had a history of clinical optic neuritis, affecting 13 eyes (65%); the remainder had electrophysiological evidence only for optic nerve involvement. One participant had been previously treated with beta-interferon for one year with treatment discontinued due to disease progression two years before recruitment to this trial. Eight healthy controls were also recruited, matched for age (mean 43 years [SD 3.5], Student's t-test, p = 0.1124) and sex (2:6 [F:M], Fisher's exact test, p = 0.618).

**Table 2:** Participant characteristics for treatment cohort

Participant	Age	Sex	MS Phenotype	Duration of MS (years)	EDSS at entry	Clinical episode of optic neuritis	Optic nerve affected	Time elapsed since first clinical episode of optic neuritis (years)
**1**	44	Male	SPMS	19	6	Yes	Right	19
**2**	51	Male	SPMS	26	6	Yes	Both	26 (left); 9 (right)
**3**	40	Female	SPMS	13	6.5	No	Left*	6
**4**	48	Male	SPMS	27	6	Yes	Left	27
**5**	48	Male	SPMS	12	6.5	Yes	Right	11
**6**	52	Male	SPMS	18	6	Yes	Right	18 & 13
**7**	53	Female	SPMS	5	6	Yes	Left	6
**8**	51	Male	SPMS	7	5.5	Yes	Both	2 (left); 7 (right)
**9**	46	Female	SPMS	11	6	Yes	Both	5
**10**	51	Male	SPMS	6	6.5	Yes	Both	6
								

SPMS = secondary progressive multiple sclerosis, EDSS = expanded Kurtzke disability status scale. * First episode of Uhthoff's phenomenon 6 years prior to recruitment.

### MSC culture results

MSCs were successfully isolated and cultured to the target dose from all bone marrow aspirates (mean total cultured dose = 2.0 ×10^6 ^cells / kg; range 1.1 to 3.7 ×10^6 ^cells / kg). Mean culture duration was 24 days (range 20 to 30 days) with a mean cell-doubling time of 1.5 days (range 1.3 to 2.0 days). All MSC cultures were characterised to ISCT definition criteria, had normal karyotype by array-CGH, and no evidence of pathogenic contamination.

### Baseline assessment results

#### (i) Patient-based measures

Baseline results for patient-based outcomes in the treatment group are shown in table 3 (table 3). Mean EDSS was 6.1 (range 5.5 to 6.5). All patients had higher MS Functional Composite (MSFC) disability scores than the National Multiple Sclerosis Society (NMSS) Task Force reference population mean (Mean MSFC z-score -1.5; range -0.4 to -5.4). Eight patients had Beck's Depression Inventory-II scores in the range for minimal depression (0 - 13), one patient scored in the range for mild depression (14 - 19), and one patient scored in the range for moderate depression (20 - 28).

**Table 3:** Patient-based measures in treatment group

	Mean	SD	Range
**BDI-II score**	8.2	7.6	1 - 25
**MSIS-29 Physical score**	66.3	9.5	51 - 80
**MSIS Psychological score**	20.7	5.2	11 - 27
			
**EDSS**	6.1	0.3	5.5 - 6.5
**ACE-R score**	92.4	5.9	85 - 100
			
**MSFC (total) z-score**	-1.5	1.4	-5.4 - -0.4
*MSFC (arm) z-score*	-1.5	0.6	-2.7 - -0.7
*MSFC (leg) z-score*	-2.0	4.1	-13.7 - -0.3
*MSFC (cognitive) z-score*	-0.9	1.1	-2.1 - 1.2
			
**Normalised brain volume (ml)**	1486	75	1361 - 1586
**T_2 _lesion volume (cm^3^)**	40.5	30.1	3.4 - 99.5
**T_1 _lesion volume (cm^3^)**	10.2	9.5	0.6 - 31.3
			

BDI-II = Beck's Depression Inventory II; MSIS-29 = Multiple Sclerosis Impact Score (29 - item); EDSS = expanded Kurtzke disability status scale; ACE-R = Addenbrooke's Cognitive Examination (Revised)

#### (ii) Optic nerve-based measures

Baseline results for optic nerve-based outcomes are shown in table 4. Visual evoked potentials were abnormal in all treatment arm participants (18 of 20 eyes showing changes consistent with demyelination). Retinal nerve fibre layer thickness was reduced by 15.3% in patients' eyes with a previous history of optic neuritis or Unthoff's phenomenon (clinically affected) compared to clinically unaffected eyes (Absolute values: 72.4 μm [SD 12.9 μm] *vs*. 85.5 μm [SD 10.2 μm]; p = 0.0399). Full-field VER latency was prolonged by 18.7% in clinically affected eyes compared to clinically unaffected eyes (Absolute values: 136.3 ms [SD 15.2 ms] *vs*. 114.8 ms [SD 7.1 ms]; p = 0.0045).

**Table 4:** Optic nerve-based measures in treatment group

	Clinically Affected (mean, SD)	Clinically Unaffected (mean, SD)	p
**Number of optic nerves (n)**	14	6	
			
**LogMAR**	0.15 (0.20)	0.15 (0.17)	0.9640
**Sloan 25%**	0.31 (0.26)	0.30 (0.20)	0.9746
**Sloan 5%**	0.66 (0.35)	0.53 (0.18)	0.4282
**Sloan 1.25%**	1.0 (0.46)	0.75 (0.15)	0.1544
			
**FM-100 Square-Root Total Error Score**	16.0 (4.4)	13.5 (2.8)	0.2188
**Visual field Mean Deviance**	-4.28 (2.04)	-3.04 (2.06)	0.2267
			
**RNFL average thickness (microns)**	72.4 (12.9)	85.5 (10.2)	0.0399
**Macular volume (mm^3^)**	6.1 (0.5)	6.6 (0.3)	0.0624
			
**Full field VER latency (milliseconds)**	136.3 (15.2)	114.8 (7.1)	0.0045
**Full-field VER amplitude (microvolts)**	4.8 (1.9)	4.5 (2.3)	0.7669
			
**Optic nerve area (mm^2^)**	8.1 (1.3)	8.9 (1.1)	0.1961
**Optic nerve MTR (pu)**	28.4 (2.9)	31.3 (2.3)	0.0515
			
**Optic nerve DTI Mean Diffusivity (×10^-3 ^mm^2^/s)**	1.17 (0.29)	1.25 (0.26)	0.5415
**Optic nerve DTI Fractional Anisotropy (×10^3^)**	298.2 (91.2)	353.3 (88.6)	0.2282
**Optic nerve DTI Radial Diffusivity (×10^-3 ^mm^2^/s)**	0.99 (0.26)	1.03 (0.24)	0.7004
**Optic nerve DTI Axial Diffusivity (×10^-3 ^mm^2^/s)**	1.53 (0.42)	1.70 (0.35)	0.4012
			

FM-100 = Farnsworth-Munsell 100-Hue test; RNFL = Retinal Nerve Fibre Layer; VER = Visual Evoked Response; DTI = Diffusion Tensor Imaging.

#### (iii) Comparative performance between patients and controls

Normalised brain volume was reduced by 11.1% in patients compared to controls (Absolute values: 1486 cm^3 ^[SD 75 cm^3^] *vs*. 1671 cm^3 ^[SD 53 cm^3^]; p < 0.00005). There were no significant differences in brain imaging MTR measures between controls and patients although there was a trend to higher MTR values in controls. Whole brain (mean) MTR was reduced by 5.3% in patients (Absolute values: 44.52 pu [SD 6.56 pu] *vs*. 47.02 pu [SD 4.48 pu]; p = 0.3976). Grey matter (mean) MTR was reduced by 7.2% in patients (Absolute values: 35.20 pu [SD 7.34 pu] *vs*. 37.95 pu [SD 5.14 pu]; p = 0.4061). White matter (mean) MTR was reduced by 4.6% in patients (Absolute values: 46.37 pu [SD 6.92 pu] *vs*. 48.63 pu [SD 4.49 pu]; p = 0.4623).

Optic nerve-based measures showed significant differences between patients and controls in measures of RNFL average thickness and optic nerve area, and in DTI measures of mean diffusivity, fractional anisotropy and radial diffusivity (table 5).

**Table 5:** Optic nerve-based measures in patients and controls

	PATIENTS		CONTROLS	
			
	Clinically Affected (mean, SD)	Clinically Unaffected (mean, SD)	(mean, SD)	p*
**Number of optic nerves (n)**	14	6	16	-
				
**RNFL average thickness (microns)**	72.4 (12.9)	85.5 (10.2)	101.6 (12.1)	0.0093
**Macular volume (mm^3^)**	6.1 (0.5)	6.6 (0.3)	6.9 (0.4)	0.1211
				
**Optic nerve area (mm^2^)**	8.1 (1.3)	8.9 (1.1)	10.3 (0.8)	0.0123
**Optic nerve MTR (pu)**	28.4 (2.9)	31.3 (2.3)	32.9 (3.4)	0.2700
				
**Optic nerve DTI Mean Diffusivity (×10^-3 ^mm^2^/s)**	1.17 (0.29)	1.25 (0.26)	0.97 (0.13)	0.0145
**Optic nerve DTI Fractional Anisotropy (×10^3^)**	298.2 (91.2)	353.3 (88.6)	530.6 (99.7)	0.0004
**Optic nerve DTI Radial Diffusivity (×10^-3 ^mm^2^/s)**	0.99 (0.26)	1.03 (0.24)	0.67 (0.13)	0.0007
**Optic nerve DTI Axial Diffusivity (×10^-3 ^mm^2^/s)**	1.53 (0.42)	1.70 (0.35)	1.58 (0.23)	0.4809
				

RNFL = Retinal Nerve Fibre Layer; VER = Visual Evoked Response; DTI = Diffusion Tensor Imaging. * Significance test for control *vs*. patient by 2-way ANOVA controlling for optic nerve status in patient group (clinically affected/unaffected)

## Discussion

The MSCIMS trial aims to establish the safety and feasibility of autologous intravenous mesenchymal stem cell therapy in multiple sclerosis. In addition, it provides an opportunity to advance trial methodology for the assessment of putative neuroprotective agents in MS, and inform the design of subsequent trials to test potential efficacy. Two aspects of design are of particular note due to their potential for wider application. First, a pre-test : post-test design was chosen in order to maximise the opportunity to inform on potential efficacy in a small cohort with an unknown effect size. Given that *intra*-individual variance in the rate of disease progression in MS measured clinically or radiologically is less than *inter*-individual variance,[23-25] the advantage of using a pre-test : post-test design is to increase the effect size between comparator groups and therefore increase statistical power by 40-80% [26]. Second, differences between patients in terms of disease pathology, course, and phenotype represent a significant challenge in MS clinical trials, necessitating the use of multidimensional outcome scales that are insensitive to small effect sizes, and serving to inflate the sample size required to achieve adequate power [27]. The MSCIMS trial uses complementary eligibility criteria and tailored outcome measures to study therapeutic response in participants who have deficits in the anterior visual pathway *as a model of wider disease processes*. This "sentinel lesion" approach forms a novel methodology for neuroprotective trials in MS and the MSCIMS trial assesses proof-of principle for its utility.

In order to establish the safety profile of the intervention, selection and timing of appropriate outcomes has been guided by the published literature on intravenous MSC therapy. Immediate adverse events consistent with type I hypersensitivity (pruritis, rash, fever) are reported in approximately 10% of subjects following intravenous administration of autologous or allogeneic MSCs. Intensive monitoring for evidence of allergic reactions is therefore scheduled around the time of infusion. Medium and long-term adverse event risks are less well characterised from the published literature, but include a theoretical risk of increased susceptibility to infection and neoplasia. Weekly assessment (×4) following infusion is designed to specifically screen for the former, and long term monitoring the latter. Given that the majority of the published cohort of patients who have undergone treatment with intravenous MSCs have been treated in the context of haematological malignancy, follow up of the MSCIMS trial cohort offers a unique opportunity to define long-term safety in a group with longer life-expectancy and a lower background rate of disease complications.

The treatment cohort in this trial is typical of patients with established progressive MS in terms of disability levels at recruitment and low relapse frequency (two participants in the treatment group experienced episodic clinical disease activity). While this group is appropriate for safety-assessment of novel therapies, it may be sub-optimal for assessment of therapeutic efficacy in later phase trials. Given that the assessment of neuroprotection requires efficacy endpoints based on demonstrating a reduction in the rate of neurodegeneration, a group showing dynamic (active) progression may be preferable in order to avoid type II error. Alternatively, in cohorts with more modest rates of neurodegeneration/progression, longer follow up may be required to achieve sufficient power. Two recent reports have described the use of intrathecally delivered autologous MSCs in MS without adverse events or significant change on global clinical outcomes [28,29]. However, this may reflect the significant challenge of demonstrating neuroprotection with global outcomes in small early phase clinical trials and a disease characterised by clinical and pathological heterogeneity. Against this background, we propose that an approach targeting a clinically articulate system with a range of tailored outcomes, as illustrated by the MSCIMS trial methodology, offers increased sensitivity to demonstrate structural and functional change in response to a putative neuroprotective intervention.

## Conclusions

The MSCIMS trial represents a novel approach for evaluating neuroprotective therapies in MS. It will establish the initial safety profile and feasibility of the intervention, allow informed design of subsequent studies to address efficacy, and test the utility of a novel methodology for neuroprotective trials in MS with potential for wider future application.

## Abbreviations

**ACE-R: **Addenbrooke's Cognitive Examination (Revised); **AD: **Axial diffusivity; **APTT: **Activated partial thromboplastin time; **BAC: **Bacterial artificial chromosome; **BDI-II: **Beck's depression inventory (II); **BOLD: **Blood oxygen level dependent; **CMV: **Cytomegalovirus; **DTI: **Diffusion tensor imaging; **EBMT: **European Group for Blood and Marrow Transplantation; **EDSS: **Expanded (Kurtzke) disability status score; **EDTA: **Ethylenediaminetetraacetic acid; **FA: **Fractional anisotropy; **fMRI: **Functional MRI; **FOV: **Field of view; **GM: **Grey matter; **ISCT: **International Society of Cellular Therapy; **JACIE: **Joint accreditation committee for ISCT-EBMT; **HIV: **Human immunodeficiency virus; **HTLV: **Human T-lymphotrophic virus; **MD: **Mean diffusivity; **MDEFT: **Modified driven equilibrium fourier transform; **MRI: **Magnetic resonance imaging; **MS: **Multiple sclerosis; **MSC: **Mesenchymal Stem Cell / Multipotent mesenchymal stromal cell; **MSCIMS: **The mesenchymal stem cells in multiple sclerosis trial; **MSFC: **Multiple sclerosis functional composite score; **MSIS-29: **Multiple sclerosis impact scale (29-item); **MTR: **Magnetisation transfer ratio; **MV: **Macular volume; **NAGM: **Normal appearing grey matter; **NAWM: **Normal appearing white matter; **OCT: **Optical coherence tomography; **PD: **Proton density; **PT: **Prothrombin time; **RD: **Radial diffusivity; **RR-MS: **Relapsing remitting multiple sclerosis; **RNFL: **Retinal nerve fibre layer; **SIENA: **Structural image evaluation using normalisation of atrophy; **SPM: **Statistical parametric mapping; **SP-MS: **Secondary progressive multiple sclerosis; **sTE fFLAIR: **Fat saturated short echo fast fluid attenuated inversion recovery; **VER: **Visual evoked response; **WB: **Whole brain; **WM: **White matter

## Competing interests

The authors declare that they have no competing interests.

## Authors' contributions

SC, DHM, DASC, & AJT were involved in the overall design of the study. DRA was involved in statistical aspects of trial design. CAWK, DJT, RSS and DLT were involved in design of the imaging sequences. MAS, CC, KR, and XLH were involved in design and execution of MSC isolation, expansion, storage and administration. AWM performed blinded assessments of visual evoked potentials. PC & MK were involved in all aspects of trial execution and manuscript preparation. RP was involved in participant assessments. MQD developed and performed CGH analysis techniques. All authors have read and approved the final version of the manuscript.
